# Ondansetron-Induced Anaphylactic Shock: An In-Depth Analysis of a Rare Adverse Event

**DOI:** 10.7759/cureus.42894

**Published:** 2023-08-03

**Authors:** Yasuyuki Suzuki, Shuang Liu, Mari Iwata, Hideshi Yamamoto, Katsuko Nishida

**Affiliations:** 1 Anesthesiology, Saiseikai Matsuyama Hospital, Matsuyama, JPN; 2 Pharmacology, Ehime University Graduate School of Medicine, Toon, JPN; 3 Dermatology, Ehime Prefectural Central Hospital, Matsuyama, JPN; 4 General Surgery, Saiseikai Matsuyama Hospital, Matsuyama, JPN

**Keywords:** anesthesia, histamine, mrgprx2, anaphylaxis, ondansetron

## Abstract

Ondansetron, a drug predominantly employed in most general anesthesia cases, is critical for mitigating postoperative nausea and vomiting prompted by anesthetics. Although infrequent side effects such as serotonin syndrome are recognized, the drug is generally acknowledged for its safety. Nonetheless, some reports showed cases of anaphylactic shock associated with ondansetron. In this context, we have meticulously analyzed an anaphylactic case triggered by ondansetron that we encountered. Our deep-dive investigation suggests that the reaction might not be a traditional type I allergic reaction, typically associated with the drug. Instead, we present the possibility that the response could be mediated through Mas-related G protein-coupled receptor X2 (MRGPRX2), a divergent pathway leading to comparatively milder symptoms of anaphylaxis. In addition to the crucial role of adrenaline in unstable hemodynamics, our case highlights the effective use of antihistamines in rapidly managing such reactions. This finding suggests a need to further examine the safety profiles of common drugs like ondansetron and the potential involvement of MRGPRX2 in drug-induced hypersensitivity reactions.

## Introduction

Ondansetron is widely recognized as possessing a low rate of adverse effects, and thus, many anesthetists are apt to employ it commonly for prophylaxis of postoperative nausea and vomiting. Nevertheless, a PubMed search reveals numerous hypersensitivity reactions [1-4]. Hence, ondansetron should be used with circumspection in the perioperative period. This report presents the details of a case that exemplifies this concern, along with immunological considerations and structural chemical analyses. Written consent was obtained from the patient himself to report the case.

## Case presentation

A 70-year-old man had recurring diverticulitis of the sigmoid colon. Consequently, a laparoscopic sigmoid colon resection was carried out under general anesthesia. His medical history included a past transfusion of concentrated red blood cells in response to bleeding caused by diverticulitis. No record of allergies was present, and all preoperative tests were normal. Additionally, there was no history of prior use of ondansetron.

The patient underwent general anesthesia, which was induced using propofol, rocuronium, and fentanyl. Prior to the commencement of the surgery, a rectus abdominis sheath block was administered using 48 ml of 0.375% ropivacaine. General anesthesia was sustained throughout the procedure with continuous intravenous infusion of remifentanil at a rate of 0.15 µg/kg/min and inhalation of 4% desflurane. Muscle relaxation was maintained using rocuronium at a 5 µg/kg/min rate.

Approximately five minutes after the ondansetron administration, the patient's systolic blood pressure suddenly dropped to 72/38 mmHg. however, there was no reflex-induced increase in heart rate, which remained at 70 bpm. General observation revealed widespread erythema on the trunk and extremities. The patient's exhaled carbon dioxide expiratory waveform did not indicate obstruction. Auscultation revealed unremarkable breath sounds, bilaterally.

**Figure 1:**
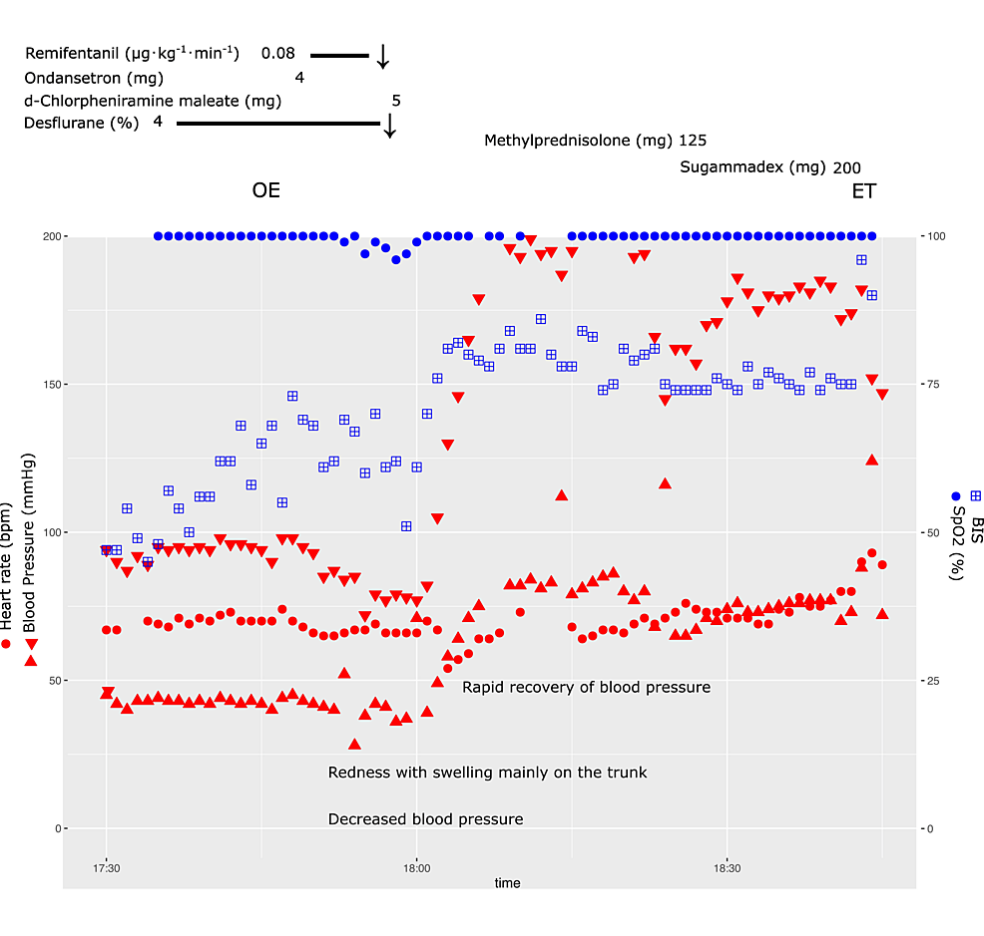
Chart of hemodynamic changes in the end stages of surgery due to an allergic reaction. Following the administration of ondansetron, a gradual decrease in blood pressure was observed, with systolic readings falling to approximately 70 mmHg. Notably, redness and swelling were primarily observed on the trunk, suggesting an allergic reaction. Upon intravenous administration of an antihistamine, a swift improvement in blood pressure was noted. Red triangle = BP; red circle = HR; blue square = BIS; blue circle above = SpO_2_. BIS indicates bispectral index; BP, blood pressure; ET, extubation; HR, heart rate; OE, operation end; SpO_2_, percutaneous arterial oxygen saturation.

We assessed the possibility of an anaphylactic shock. However, we disregarded the necessity for a prompt intramuscular adrenaline injection. Instead, we administered a 5 mg intravenous dose of the antihistamine d-chlorpheniramine maleate, rapidly improving blood pressure within a few minutes (Figure 1). Intravenous methylprednisolone (125 mg) was administered to prevent a late allergic reaction. Blood was collected to determine tryptase and histamine right after the onset of symptoms, and serum was promptly separated at 4°C and stored at -45°C.

The generalized skin eruption was mild, and the patient emerged from anesthesia smoothly. The patient was extubated without incident. The patient was observed overnight in the critical care unit for meticulous monitoring. Serum samples were extracted the day after surgery. The patient had a successful postoperative course and was discharged ten days after surgery.

Following the perioperative anaphylaxis guidelines of the Japanese Society of Anesthesiologists, skin tests for the suspect drugs were performed five weeks after the surgery. Rocuronium, acetaminophen, and ondansetron were tested. Rocuronium was discontinued for continuous administration 30 minutes before the end of surgery. Although it is unlikely to cause this reaction, we decided to perform skin testing since it is considered one of the leading causative agents of perioperative anaphylaxis.

Both prick and intradermal tests did not satisfy the criteria and were negative. Nevertheless, only the intradermal test result for ondansetron revealed slightly augmented erythema (Table 1), and although it did not satisfy the criteria, ondansetron was deemed the likely inciting agent. On the day of surgery, immediate serum analysis revealed a tryptase level of 1.9 µg/L (with a normal range being 2.1 to 9.0 µg/L) and a histamine level of 17.2 ng/ml (with a normal range being 0.15 ng/ml to 1.23 ng/ml).

**Table 1:** Skin test results of the suspected causative agent. Ondansetron was associated with more hypersensitivity reactions than the other drugs, although to a degree that did not meet the diagnostic criteria.

Substance	Test type	Concentration	Erythema size (mm)	Wheal response
Ondansetron (2mg/ml)				
	Prick	As is	6 × 5	No
	Prick	1/10	3 × 2	No
	Intradermal	1/100	9 × 8	No
Rocuronium (10 mg/ml)				
	Prick	As is	3 × 2	No
	Prick	1/10	4 × 2	No
	Intradermal	1/100	4 × 3	No
Acetaminophen (10 mg/ml)				
	Prick	As is	3 × 2	No
	Prick	1/10	4 × 3	No
	Intradermal	1/100	3 × 2	No
Histamine (10 mg/ml)				
	Prick	As is	32 ×32	No
Saline				
	Prick	As is	5 × 6	No

**Figure 2:**
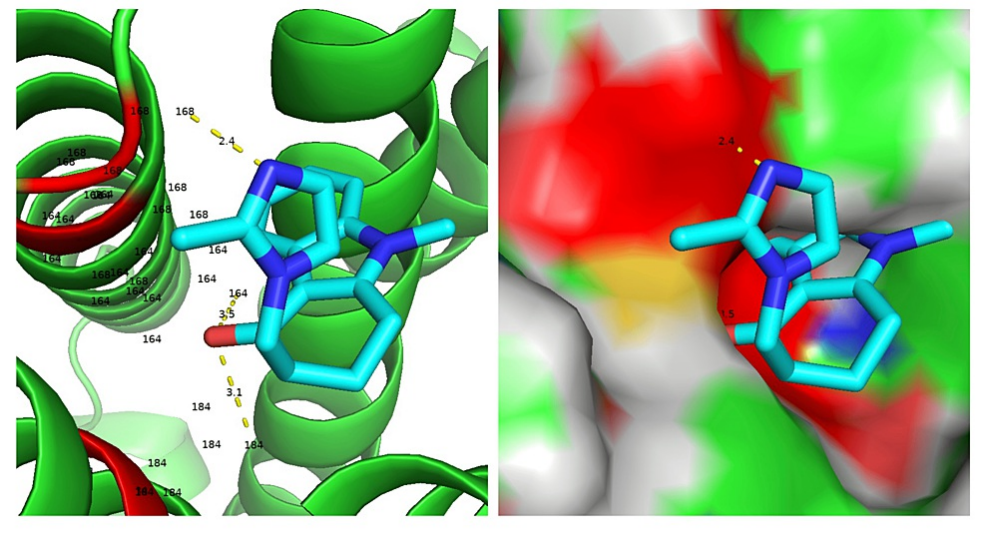
AutoDock Vina-derived interactions of ondansetron with MRGPRX2 agonist binding sites. Previous research suggested that E164 and D184 might be critical in binding to compound 48/80, a Mas-related G protein-coupled receptor X2 (MRGPRX2) agonist. Docking studies using AutoDock Vina with ondansetron have computationally demonstrated interactions with analogous amino acids. These in silico results also propose that ondansetron could stimulate MRGPRX2, prompting histamine release.

Given that no tryptase was secreted and only histamine levels were elevated, we considered the potential for ondansetron to stimulate the Mas-related G protein-coupled receptor X2 (MRGPRX2), as opposed to inducing a type I hypersensitivity reaction [5]. Utilizing compound 48/80, a potent MRGPRX2 agonist, along with structural data on ondansetron retrieved from PubChem ID 4595, we generated a Protein Data Bank (PDB) file containing information on hydrogen atom loading and charge using Chimera version 1.17.1. The three-dimensional structure data for MRGPRX2 used in docking analyses was downloaded from https://www.rcsb.org/ with ID: 7VV6. Both the receptor and ondansetron were prepared for docking by adding hydrogen atoms and charges using Chimera. Docking analyses were subsequently conducted using AutoDock Vina version 1.1.2 [6]. In performing the docking experiments, we specified the region around the binding pocket, based on electron microscopy data where C48/80 is docked onto MRGPRX2 [7]. Our results indicated that ondansetron interacts with residues D164 and E184 of MRGPRX2 (Figure 2), consistent with previous reports [7]. These two amino acids are particularly significant as it is reported that altering either one results in decreased responsiveness to the agonist, suggesting a possible stimulation of MRGPRX2 by ondansetron when interactions occur at these sites.

## Discussion

In the past, ondansetron has been extensively used for combating nausea resulting from chemotherapy for malignant tumors. Chemotherapy courses are often repeated, and ondansetron may have been administered on each occasion, potentially inducing a state of sensitization. In such cases of sensitization, with the production of specific immunoglobulin E (IgE) antibodies, anaphylactic reactions may transpire. Nevertheless, the cases we have encountered have yet to be exposed to ondansetron. Thus, it is unlikely that the patient had specific IgE antibodies due to sensitization by ondansetron.

It is well-documented that compounds with similar chemical structures can induce the production of specific IgE antibodies, leading to allergic reactions. In anesthesiology, it has been reported that patients can become sensitized to cosmetics containing quaternary amines and display hypersensitivity reactions to rocuronium [8]. If compounds sharing a similar structure to ondansetron are widely used in everyday life, this could be one of the potential causes, though the exact culprit remains unclear.

Worse, we need help testing whether this patient has specific IgE antibodies to ondansetron. Specific IgE antibody testing for ondansetron is possible with ImmunoCAP. However, it costs hundreds of thousands of dollars as the test kits need to be custom-made, and the test cannot be performed in Japan and must be sent to Europe. It is difficult to establish that a specific IgE antibody-mediated type I allergic reaction is involved in this case. In addition, as shown for other drugs, specific IgE antibody tests are often false-negative [9], and in some cases, even if the test is performed, it cannot be definitive.

As an allergic reaction not mediated by specific IgE antibodies, the pathway in which drugs directly stimulate MRGPRX2 on mast cells, resulting in the release of histamine, has attracted attention. A wide spectrum of medicines may stimulate MRGPRX2 to release histamine, including rocuronium, morphine, and levofloxacin [10-12].

The clinical presentation of this case suggests that the observed reaction may have been mediated by MRGPRX2 rather than a conventional type I allergic reaction. Whereas type I allergies trigger complete mast cell degranulation, releasing a surge of inflammatory cytokines, the MRGPRX2-mediated response may follow a "kiss and run" pattern [5]. This involves a finely tuned process of granule and cell membrane fusion and detachment, leading to partial degranulation. Consequently, MRGPRX2-mediated reactions may present less severely than type I allergic reactions. In the context of the current case, it's important to clarify that the patient experienced only mild hypotension, with systolic blood pressure around 70mmHg, rather than persistent severe hypotension. This and minor skin symptoms confined to the trunk indicate that the hypersensitivity reaction was not immediately life-threatening. The relatively mild response might be due to "kiss and run" degranulation mediated by MRGPRX2, rather than full degranulation, resulting in the gradual release of low molecular weight histamine, which aligns with our detection of solely low molecular weight histamine without any high molecular weight tryptase. This bolsters our hypothesis and is consistent with our observations. 

Moreover, in silico docking studies revealed that ondansetron, like the potent MRGPRX2 agonist compound 48/80, interacts with MRGPRX2 [7]. This finding implicates MRGPRX2 as a potential facilitator of the hypersensitivity reaction observed in this case.

We have an experimental platform to investigate histamine release in rat basophil-like cells RBL-2H3 expressing MRGPRX2 [13]. We plan to investigate whether MRGPRX2-mediated responses occur in cultured cells when adding ondansetron. If the involvement of MRGPRX2 is clarified, ondansetron may become the focus of attention as a substance prone to perioperative hypersensitivity reactions.

Recently, the basophil activation test has gained attention as a method to investigate the causes of drug hypersensitivity reactions. This technique involves the collection of basophils from the patient's blood, which are then exposed to the suspected substance, and the expression of surface markers such as CD63 is observed using flow cytometry. Different surface markers might be expressed depending on the degranulation pattern, making this method potentially useful for analyzing reactions mediated by IgE antibodies or MRGPRX2. However, a significant drawback is that live basophils are required, necessitating the test to be performed within a few hours after blood collection. Furthermore, the necessity for a nearby lab equipped with a flow cytometer means this test is not easily accessible, limiting its use as a standard examination at present.

One limitation of this report is that we cannot conclusively rule out the possibility that causes other than hypersensitivity reactions may have contributed to the drop in blood pressure. As we were in the late stages of surgery and invasive stimuli decreased, the relative effects of remifentanil and inhaled anesthetics may have intensified, potentially leading to hypotension. While this should be noted, the timing of the ondansetron administration, the presence of a mild cutaneous reaction, and the confirmed release of histamine lead us to attribute the hypotension primarily to a hypersensitivity reaction.

We also want to consider the role of antihistamines in treating this relatively mild and nonfatal form of anaphylaxis, as seen in this case. In this case, the shock state improved rapidly with intravenous diphenhydramine. The basis of treatment for anaphylaxis shock involves maintaining both respiratory function and circulatory function. As many guidelines suggest, the appropriate administration of adrenaline should not be delayed [14]. However, we emphasize the importance of rapidly distributing antihistamines, which are considered a relatively low priority, to address both respiratory and circulatory aspects, by giving them intravenously rather than orally. This is because the start of anaphylaxis is attributed to histamine. Histamine, which stimulates histamine receptor H1 in the vascular endothelium, exhibits potent vasodilatory effects. It may be essential to promptly suppress this vasodilatory effect by rapidly administering antihistamines, potent competitive inhibitors. It should be noted that, while the general side effects of antihistamines include anticholinergic effects and sedation, serious side effects are not common. However, caution may be necessary with the classical antihistamine, clemastine, which is not widely used currently, as there have been reports of severe bradycardia and hypotension.

## Conclusions

In conclusion, the reported patient case adds weight to the hypothesis that ondansetron could stimulate MRGPRX2, rather than triggering a conventional IgE-mediated type I allergic reaction. The MRGPRX2-mediated pathway potentially presents less severe symptoms and explains the observed patient condition. The need to ascertain this cause-effect relationship warrants an expensive specific IgE antibody test, which may not even provide definitive results. While it remains unquestionable that adrenaline should not be hesitated in unstable hemodynamic conditions, our case highlights the potentially critical role of immediate antihistamine treatment, given their capacity to rapidly suppress the vasodilatory effect of histamine, hence potentially playing a significant role in managing anaphylactic reactions.
